# Advancements in the Treatment Landscape of Hepatocellular Carcinoma

**DOI:** 10.3390/cancers16051054

**Published:** 2024-03-05

**Authors:** Takefumi Kimura

**Affiliations:** 1 Department of Medicine, Division of Gastroenterology and Hepatology, Shinshu University School of Medicine, Asahi 3-1-1, Matsumoto 390-8621, Japan; t_kimura@shinshu-u.ac.jp; 2 Consultation Center for Liver Diseases, Shinshu University Hospital, Matsumoto 390-8621, Japan

The landscape of hepatocellular carcinoma (HCC) treatment has expanded significantly with the advent of multi-kinase inhibitors and immune checkpoint inhibitors [1]. In addition to drug therapy, there have been remarkable advances in liver transplantation, minimally invasive surgery, the technology involved in ablation therapy, the choice of embolization materials and anticancer drugs for embolization therapy, and radiation therapy [2,3]. Modern HCC treatment requires not only the curative treatment of early-stage lesions but also the establishment of new treatment systems that lead to a cure for advanced HCC and appropriate treatment of recurrent HCC. In addition, the number of non-alcoholic steatohepatitis (NASH)/metabolic dysfunction-associated steatohepatitis (MASH) and alcoholic liver cancers are expected to increase as viral hepatitis is controlled [4]. The management of side effects in these non-viral HCCs may differ due to differences in drug sensitivity, accompanied by diabetes mellitus and hypertension. In this Special Issue, we showcase five articles that not only elucidate various facets of advanced HCC treatment but also provide actionable insights into optimizing patient care.

The first article included in this Special Issue, based on a retrospective real-world cohort study, evaluates the safety and efficacy of trans-arterial radioembolization (TARE) with Yttrium-90 (Y90) versus systemic combination therapy with atezolizumab plus bevacizumab in patients with advanced HCC. Among the 74 patients included, the study reports a median overall survival of 7.1 months with TARE and 13.0 months with systemic combination therapy, hinting at a potential survival benefit favoring the latter (*p* = 0.07). In patients with underlying cirrhosis, baseline liver function was shown to be a predictor of radioembolization-induced liver disease (REILD), underlining the importance of individualized treatment strategies in the management of advanced HCC. In the realm of actual HCC practice, multiple treatment modalities are often combined [5]. Following the findings of this study, a newfound interest lies in exploring the efficacy and safety of combining TARE with Y90 alongside systemic therapies such as atezolizumab plus bevacizumab.

In the second article included in this Special Issue, researchers explore the synergistic effects of cabozantinib and cannabidiol (CBD) in enhancing the sensitivity of HCC cells to anticancer activity, mainly through cellular experiments. Their findings reveal a significant augmentation of cytotoxicity and apoptosis in HCC cells upon combination treatment, attributed primarily to p53 phosphorylation regulated by endoplasmic reticulum (ER) stress. These mechanistic insights hold promise for the development of novel combination therapies targeting HCC, as evidenced by the compelling data presented. Notably, sorafenib, lenvatinib, and regorafenib showed weak or no synergistic anticancer susceptibility-enhancing effects with CBD.

NASH/MASH has emerged as a prominent precursor to HCC, yet effective therapeutic interventions remain elusive [6]. The progress in this field depends on the availability of reliable preclinical models that show the steady progression to NASH/MASH, liver cirrhosis, and HCC [7]. The third article included in this Special Issue introduces a novel NASH-HCC mouse model characterized by rapid progression from NASH to liver cirrhosis and ultimately HCC. A newly developed choline-deficient and methionine-restricted high-fat diet (OYC-NASH2 diet) was administered to male C57BL/6J mice, which showed marked lipidosis, lobular inflammation, and fibrosis, histologically diagnosed as NASH, only 3 weeks after administration. Furthermore, liver nodules emerged at 12 weeks of treatment, >2 mm diameter liver tumors developed in all mice at 24 weeks of treatment, and HCC appeared after 36 weeks of treatment. This novel mouse model of NASH-HCC may be useful for studying the pathogenesis of human NASH-HCC and for developing novel therapeutic agents.

Although several drugs, including immune checkpoint inhibitors, are now available for the treatment of HCC, the objective response rate for these drugs is currently only 30–40% [8,9]. Furthermore, treatment barriers due to drug-induced side effects cannot be ignored. Therefore, there is a need to identify new predictive biomarkers and improve our knowledge of the mechanisms of response or resistance to systemic chemotherapy. The fourth paper included in this Special Issue outlines an extensive study conducted on the potential of HCC biomarkers from blood, tissue, and imaging information that can be used in practice to predict the response to systemic therapy before its initiation. Currently, the most important factor for predicting the efficacy of immune checkpoint inhibitors in HCC is to accurately assess the state of T-cell immunity in the tumor microenvironment before treatment. This paper states that assessing the tumor microenvironment and immune environment is more achievable than assessing tumor cell diversity and heterogeneity [10]. Specifically, the recommendation to collect tumor tissue and assess CD8+ T-cell infiltration is very valuable guidance. Furthermore, it is imperative to acknowledge the overarching objective of assessing the immune microenvironment through the use of serum markers and imaging in the future, thereby eliminating the necessity for tumor tissue collection. This advancement holds the promise of accurately predicting therapeutic response, thereby enhancing patient care and treatment outcomes.

Finally, in the fifth report included in this Special Issue, a systematic review and meta-analysis were conducted to determine the prevalence of reduced skeletal muscle mass (LSMM) and its impact on treatment outcomes in HCC patients receiving systemic therapy. The pooled analysis highlighted a significant association between LSMM and poor overall survival (HR, 1.70) and poor progression-free survival (HR, 1.32). Subgroup analyses by type of systemic therapy (sorafenib, lenvatinib, and immunotherapy) showed similar results. The clinical significance of addressing LSMM in the management of advanced HCC was highlighted.

In conclusion, this Special Issue underscores the dynamic nature of HCC treatment and the relentless pursuit of therapeutic innovation aimed at improving patient outcomes. Through interdisciplinary collaboration and translational research efforts, we endeavor to translate scientific discoveries into tangible clinical benefits for patients battling advanced HCC. 

## References

[1] Wong K.M., King G.G., Harris W.P. (2022). The Treatment Landscape of Advanced Hepatocellular Carcinoma. Curr. Oncol. Rep..

[2] Llovet J.M., Castet F., Heikenwalder M., Maini M.K., Mazzaferro V., Pinato D.J., Pikarsky E., Zhu A.X., Finn R.S. (2022). Immunotherapies for hepatocellular carcinoma. Nat. Rev. Clin. Oncol..

[3] Tumen D., Heumann P., Gulow K., Demirci C.N., Cosma L.S., Muller M., Kandulski A. (2022). Pathogenesis and Current Treatment Strategies of Hepatocellular Carcinoma. Biomedicines.

[4] Kimura T., Kobayashi A., Tanaka N., Sano K., Komatsu M., Fujimori N., Yamazaki T., Shibata S., Ichikawa Y., Joshita S. (2017). Clinicopathological characteristics of non-B non-C hepatocellular carcinoma without past hepatitis B virus infection. Hepatol. Res..

[5] Angeli-Pahim I., Chambers A., Duarte S., Zarrinpar A. (2023). Current Trends in Surgical Management of Hepatocellular Carcinoma. Cancers.

[6] Tanaka N., Kimura T., Fujimori N., Nagaya T., Komatsu M., Tanaka E. (2019). Current status, problems, and perspectives of non-alcoholic fatty liver disease research. World J. Gastroenterol..

[7] Wang X., Tanaka N., Hu X., Kimura T., Lu Y., Jia F., Sato Y., Nakayama J., Moriya K., Koike K. (2019). A high-cholesterol diet promotes steatohepatitis and liver tumorigenesis in HCV core gene transgenic mice. Arch. Toxicol..

[8] Abou-Alfa G.K., Lau G., Kudo M., Chan S.L., Kelley R.K., Furuse J., Sukeepaisarnjaroen W., Kang Y.K., Van Dao T., De Toni E.N. (2022). Tremelimumab plus Durvalumab in Unresectable Hepatocellular Carcinoma. NEJM Evid..

[9] Finn R.S., Qin S., Ikeda M., Galle P.R., Ducreux M., Kim T.Y., Kudo M., Breder V., Merle P., Kaseb A.O. (2020). Atezolizumab plus Bevacizumab in Unresectable Hepatocellular Carcinoma. N. Engl. J. Med..

[10] Qin R., Jin T., Xu F. (2023). Biomarkers predicting the efficacy of immune checkpoint inhibitors in hepatocellular carcinoma. Front. Immunol..

